# Mg or Zn for Ca substitution improves the sintering of bioglass 45S5

**DOI:** 10.1038/s41598-020-72091-7

**Published:** 2020-09-29

**Authors:** R. Wetzel, M. Blochberger, F. Scheffler, L. Hupa, Delia S. Brauer

**Affiliations:** 1 Otto Schott Institute of Materials Research, Friedrich Schiller University, Fraunhoferstr. 6, 07743 Jena, Germany; 2 Johan Gadolin Process Chemistry Centre, Åbo Akademi University, Piispankatu 8, 20500 Turku, Finland

**Keywords:** Glasses, Biomaterials

## Abstract

Bioglass 45S5 is well-known for its bioactivity, but it possesses poor sintering behaviour owing to viscous flow being inhibited by the crystallisation of sodium calcium silicate phases. Mg or Zn were partially (0, 25, 50, 75%) or fully (100%) substituted for Ca on a molar base, and thermal properties (differential scanning calorimetry, dilatometry) and sintering (heating microscopy, SEM and X-ray diffraction) were investigated. Here we show that sintering can be improved significantly by partial or complete substitution of Mg or Zn for Ca, owing to a pronounced decrease in crystallisation tendency. Glass transition temperature and dilatometric softening point went through minima for mixed compositions, with random mixing of Mg/Ca or Zn/Ca ions in the glass structure and the resulting effect on configurational entropy being a likely explanation. As the onset of crystallisation did not vary much with substitution, substituted glasses possessed a wider temperature range for sintering, resulting in up to 57% and 27% sample height reduction for Mg and Zn substituted glasses, respectively, compared to only 3% height reduction for Bioglass 45S5. Taken together, these results suggest that using a combination of modifiers, particularly alkaline earths or zinc, may be a promising approach for improving the sintering of Bioglass 45S5.

## Introduction

Bioglass 45S5 degrades in contact with physiological solutions, releases ions and forms a biomimetic apatite surface layer^1^. These properties are key to the clinical success of Bioglass, as after implantation into a bone defect they allow for bone bonding^2^ as well as for complete degradation of the glass, to be ultimately replaced by the patient's own natural bone^3^. These degradation and bone bonding properties of 45S5 are typical characteristics of a bioactive material^2^. The pronounced bioactivity of Bioglass 45S5 has its origin in its atomic set-up^4,5^: compared to more common silicate glasses, such as window glass, Bioglass contains larger amounts of modifier oxides, resulting in relatively large numbers of non-bridging oxygen atoms, which disrupt the silicate network and lower its polymerisation^6^.

This highly disrupted glass network also leads to a pronounced tendency to crystallise and thus limits high temperature processing such as fibre drawing^7^ or sintering^8^. For bioactive glass applications, particularly in bone regeneration, sintered complex, three-dimensional porous scaffolds would be of great interest as porous implant materials, simulating the structure of trabecular bone and guiding in-growing cells, or as templates in tissue engineering^3^. Crystallisation of Bioglass occurs close to glass transition and therefore impedes sintering by preventing viscous flow^9^. As a result, scaffolds based on Bioglass typically are at least partially crystalline and often suffer from poor mechanical properties^10^.

To improve sintering, incorporation of magnesium ions has been studied, typically replacing calcium ions^11,12^. The beneficial effect of magnesium incorporation on glass processing is typically explained by their higher field strength compared to calcium ions^13^. As zinc ions have a field strength similar to that of magnesium ions, a similarly beneficial effect can be expected. Their beneficial biological action in the human body makes Mg and Zn ions of additional interest as components in bioactive glasses. Both ions are co-factors in various enzymes^14,15^ and known for their key roles in bone formation and mineralisation^16–18^.

The aim of this study was therefore to investigate the effect of systematic Mg or Zn for Ca substitution on the thermal properties of Bioglass 45S5. Special emphasis was put on characterising glass processing, particularly crystallisation tendency and sintering, using a combination of thermal analysis, heating microscopy, X-ray diffraction and electron microscopy.

## Results and discussion

**Figure 1:**
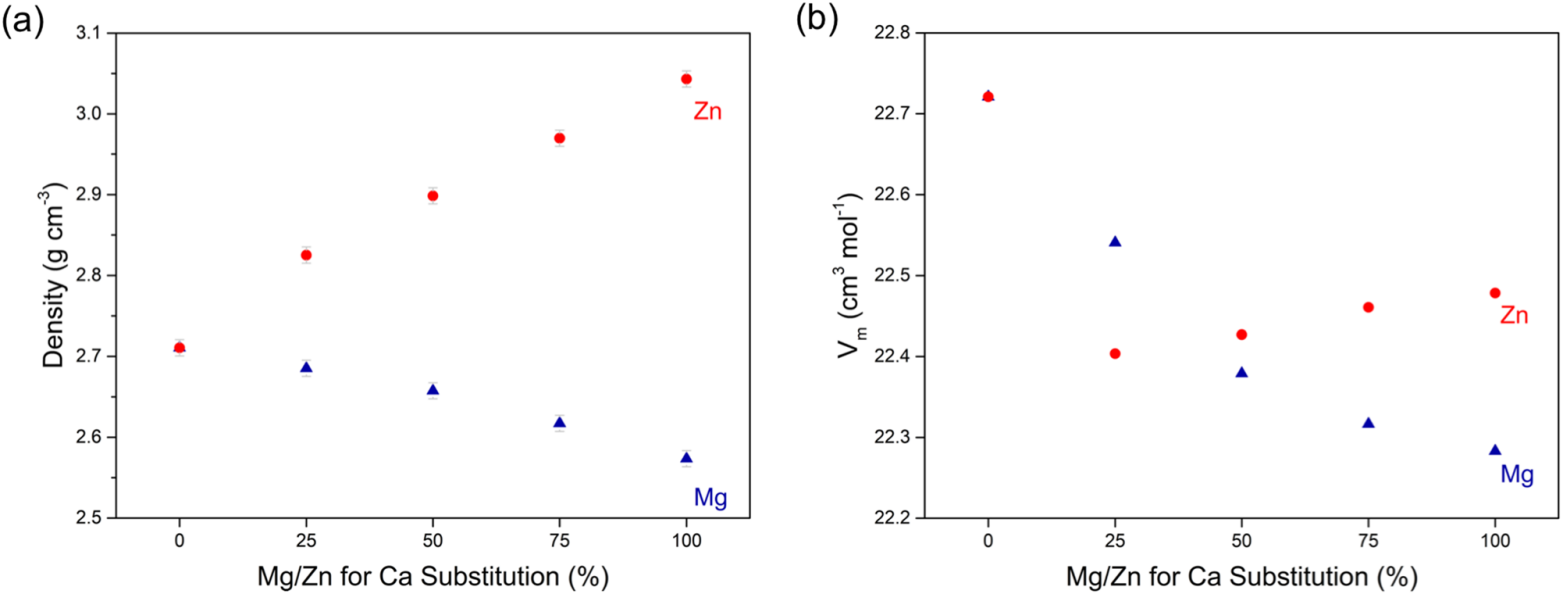
(**a**) Density and (**b**) molar volume (V_m_) of Mg/Zn substituted Bioglass 45S5.

Glasses were obtained in an X-ray amorphous state as shown previously^19^. Glass density decreased with Mg for Ca substitution (Fig. 1a) owing to the lower atomic weight of magnesium compared to calcium, while Zn substitution caused a density increase owing to its higher atomic weight. Molar volume (V_m_; Fig. 1b) showed a continuous decrease with Mg substitution (Fig. 1b), owing to the smaller ionic radius^20^ of Mg^2+^ (57 pm) compared to Ca^2+^ (100 pm). This trend suggests the glass network to become continuously more compact, as shown previously for Mg for Ca substitution or other substitutions of smaller modifier ions^21–24^. Although Zn^2+^ has an ionic radius (60 pm) similar to that of Mg^2+^, Zn substitution resulted in a different trend: a huge drop in V_m_ was observed for 25% Zn for Ca substitution (Fig. 1b), while increasing substitution seemed to cause a slight V_m_ increase. This suggests that zinc affects the glass network differently compared to magnesium. Molecular dynamics simulations by Lusvardi et al.^25^ suggested that Zn entered the silicate network as ZnO_4_ tetrahedra with longer cation–oxygen distances than for SiO_4_ tetrahedra. This was caused by sodium ions clustering around the ZnO_4_ tetrahedra for charge-compensation and may be an explanation for the small network expansion observed here for substitutions above 25% as well as for the observed effect of Zn for Ca substitution on ion release from Bioglass 45S5, published earlier^19^. Although a similar effect has been suggested for magnesium substitution^21^, it is not supported by the data presented here, by ion release data from Mg substituted Bioglass^19^ nor by molecular dynamics (MD) simulations published in the literature^26^.

**Figure 2:**
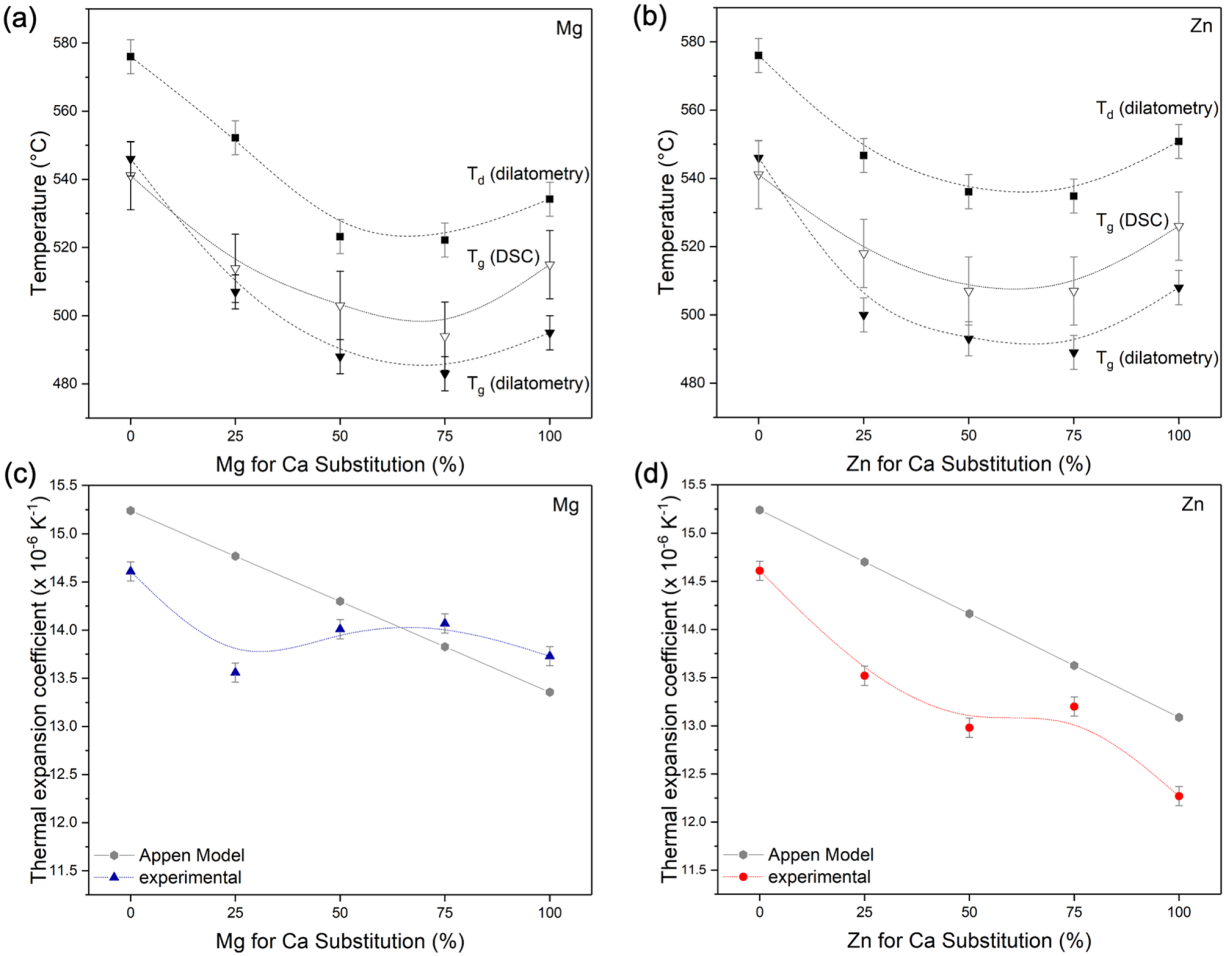
(**a**, **b**) Glass transition temperature (T_g_) obtained from DSC and dilatometry as well as dilatometric softening point (T_d_) of the (**a**) Mg and (**b**) Zn series. (**c**, **d**) Experimental and calculated thermal expansion coefficient (TEC) for the (**c**) Mg and (**d**) Zn series. (Lines are visual guides only.)

T_g_ values obtained from DSC or dilatometry (Fig. 2a,b) differed by up to 20 K, but results were comparable for Mg and Zn substituted glasses. T_g_ and T_d_ showed comparable trends for both series, decreasing initially upon substitution, as recently shown for low substitutions^27^, and reaching a minimum for substitutions of about 50 to 75% before increasing again for the fully substituted compositions. Based on ionic radius differences between Ca^2+^ and Mg^2+^ or Zn^2+^, a continuous increase in T_g_ or T_d_ would have been expected with Mg or Zn substitution, owing to their larger field strength^13^. Such minimum trends in properties involving ion transport, e.g. ionic conductivity, thermal properties or ion release, are typical for glasses containing two types of alkali metal oxide, displaying the well-known mixed alkali effect^22,28,29^, and Kjeldsen et al. described a mixed alkaline earth effect in calcium/magnesium-containing aluminosilicate glasses^30^. Such non-linear changes in thermal properties have also been observed previously for mixed Ca/Mg silicate and aluminosilicate glasses^31^, where viscosity near T_g_ showed minimum trends. This was explained by random mixing of calcium and magnesium ions in the glass as suggested by calculations of the configurational entropy, which also underwent non-linear trends with increasing substitution. The random mixing of Mg^2+^ and Ca^2+^ was later confirmed by solid-state NMR experiments^32^. The similar trends in T_g_ and T_d_ for Mg and Zn substituted glasses not only suggest random mixing of Mg^2+^ and Ca^2+^ in the present glass system, but also a similar behaviour of the two ions, i.e. random mixing, for Zn^2+^ and Ca^2+^. It is interesting to note that T_g_ and T_d_ of the fully Mg or Zn substituted glasses are lower than values observed for Bioglass 45S5, despite the higher field strength of Mg^2+^ and Zn^2+^. This may be related to the presence of an additional modifier cation, Na^+^, in the glass system and the resulting ion mixing or preferential bonding. Indeed, a trend of a magnesium and sodium-containing glass having a lower T_g_ than the corresponding calcium composition has been observed previously^33^. By contrast, corresponding compositions containing a different alkali modifier (lithium, potassium or caesium) showed the expected behaviour, i.e. the calcium composition having a lower T_g_ than the corresponding magnesium composition^33^.

Thermal expansion coefficients decreased with increasing Mg or Zn for Ca substitution (Fig. 2c,d). This is likely to be caused by the smaller ionic radii, i.e. their higher field strength, of Mg^2+^ and Zn^2+^ compared to Ca^2+^. Calculated Appen TEC values confirmed this trend, as it uses significantly lower factors for MgO (5) and ZnO (6) compared to CaO (13)^34^. While TEC values based on the Appen model were comparable, experimental thermal expansion showed some scattering and decreased much more for Zn glasses than Mg glasses. In addition, experimental TEC results were lower than those calculated based on Appen factors. The Appen model uses empirical factors for different oxides obtained from various soda-lime silicate glasses. The glasses in the present study are multi-component glasses, containing phosphate in addition to silicate, which could possibly explain the deviation between experimental and calculated TEC values^11^.

**Figure 3:**
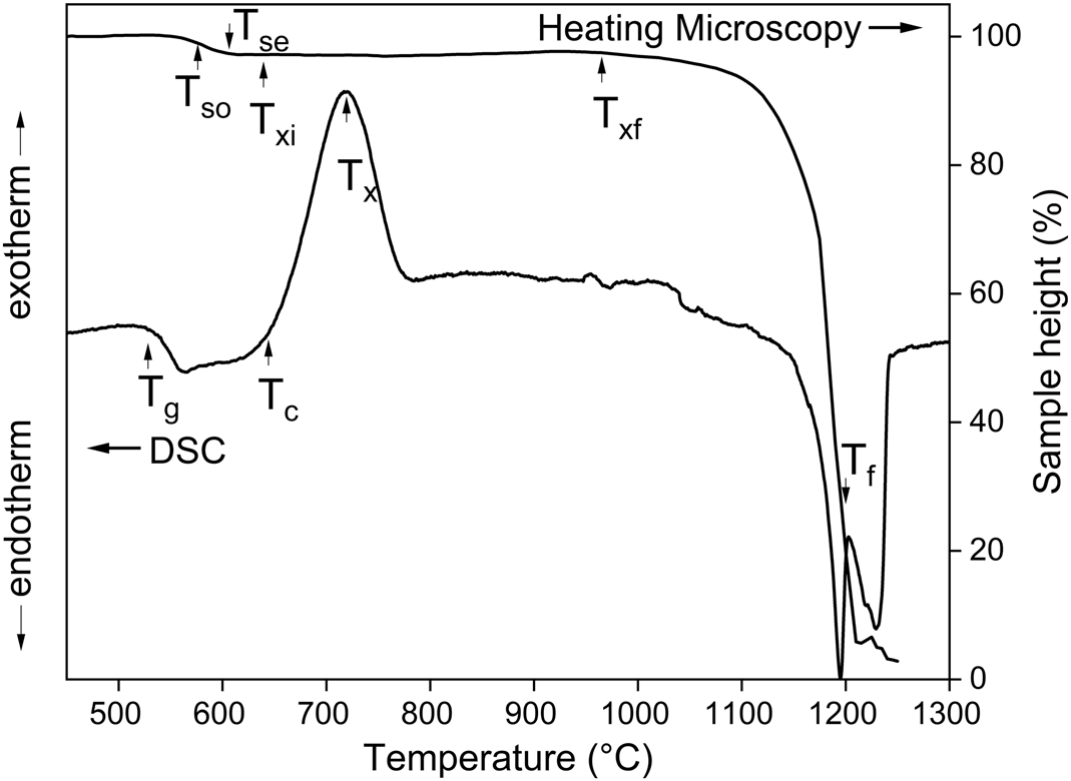
DSC trace (left axis) and heating microscopy curve (right axis) of Bioglass 45S5. *T*_*so*_ beginning of sintering, *T*_*se*_ end of sintering, *T*_*xi*_* to T*_*xf*_ crystallisation domain, *T*_*g*_ transition temperature, *T*_*c*_ crystallisation onset, *T*_*x*_ crystallisation peak, *T*_*f*_ liquidus temperature.

**Figure 4:**
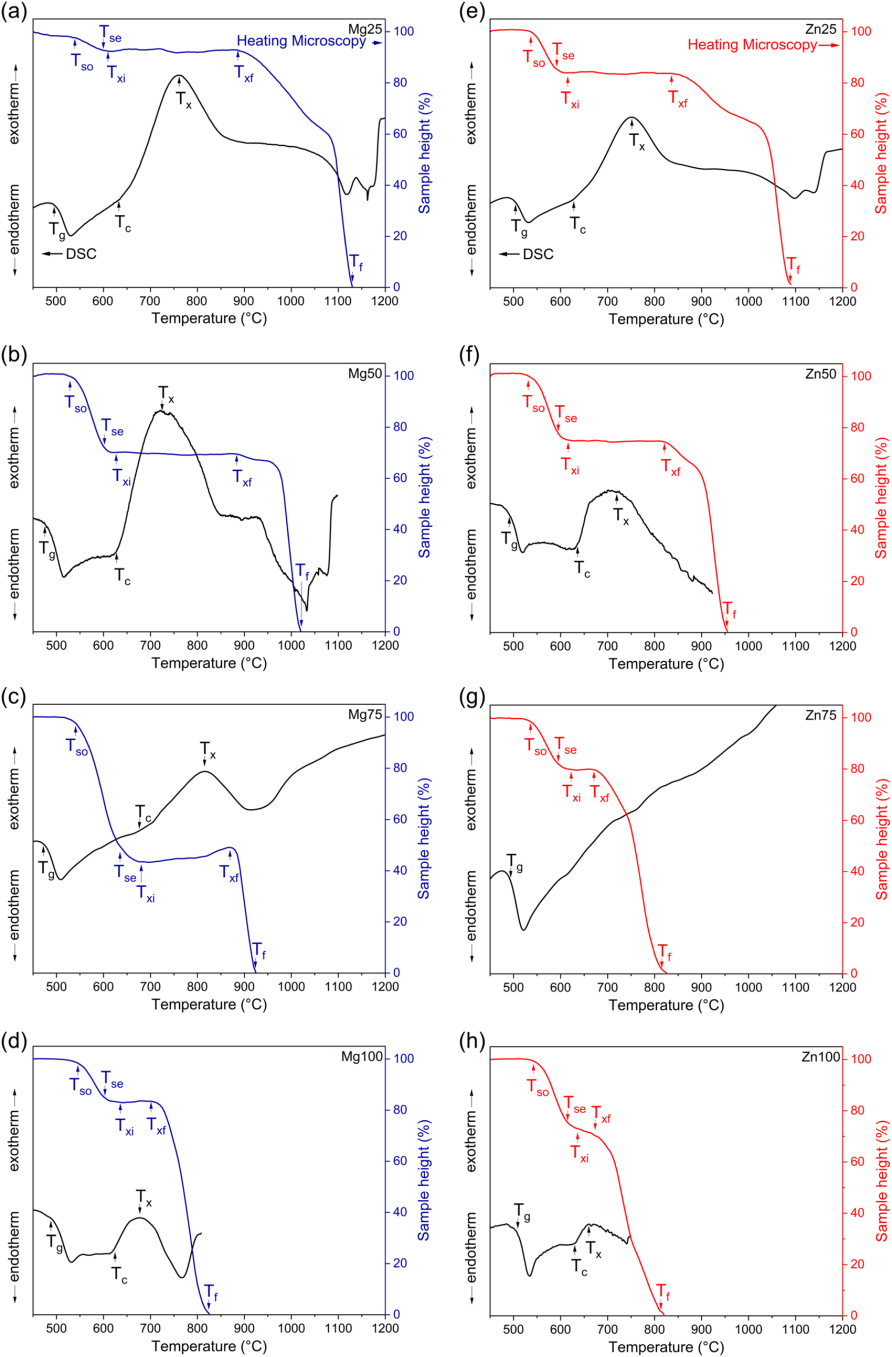
DSC traces (left axes) and heating microscopy curves (right axes) for (**a**–**d**) Mg and (**e**–**h**) Zn substituted glasses. *T*_*so*_ beginning of sintering, *T*_*se*_ end of sintering, *T*_*xi*_* to T*_*xf*_ crystallisation domain, *T*_*g*_ transition temperature, *T*_*c*_ crystallisation onset, *T*_*x*_ crystallisation peak, *T*_*f*_ liquidus temperature.

We present DSC traces superimposed on heating microscopy sintering curves of glass 45S5 (Fig. 3) as well as for Mg and Zn glasses (Fig. 4). The DSC trace of 45S5 shows a narrow crystallisation peak from 650 to 780 °C and distinct endothermic effects from just below 1,200 °C. The DSC traces for the Mg and Zn containing glasses exhibit similar trends, except that some of the Zn substituted glasses did not show such clear crystallisation peaks. Generally, the onset of crystallisation varied relatively little with substitution.

**Figure 5:**
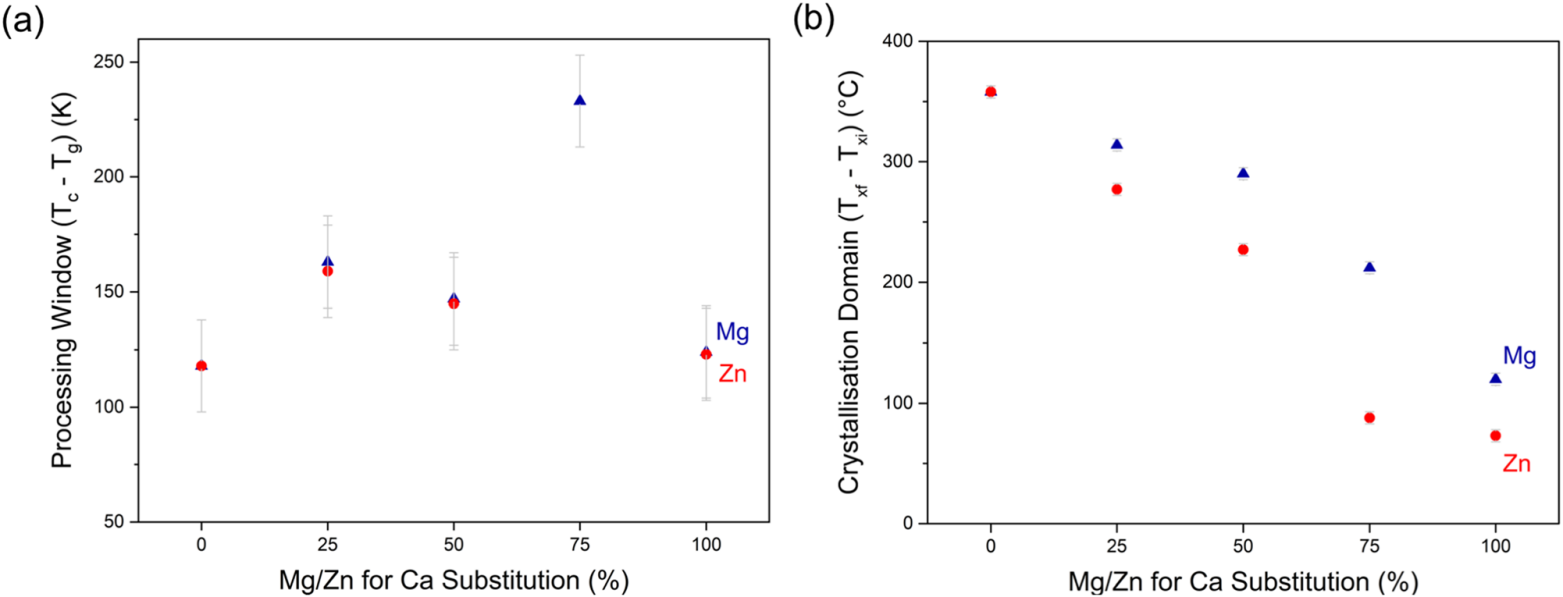
(**a**) Processing window (T_c_–T_g_) and (**b**) crystallisation domain (T_xf_–T_xi_) vs. substitution.

DSC data can give information on the high temperature processing properties of glasses. Typical parameters for describing glass processing include the processing window, i.e. the temperature range between T_g_ and the onset of crystallisation, and various glass stability parameters, which also take the liquidus temperature into account (see e.g. the review paper by Nascimento et al.^35^). As liquidus temperatures were obtained for glasses with the lowest substitution only, we refrained from calculations of glass stability parameters and evaluated the high temperature processing behaviour by other means, as described below. Where crystallisation exotherms could be identified, the processing window was calculated, and results are presented in Fig. 5a. Bioglass 45S5 is known for its narrow processing range (processing window 118 K)^8,36^, while Mg substitution resulted in a widening of the processing window. Interestingly, the processing windows of glasses Mg25 and Mg50 did not differ much within the error limits; only Mg75 showed a much wider processing window (233 K). The processing window of the fully Mg substituted glass was similar to unsubstituted Bioglass 45S5 (124 K). Processing windows of Zn substituted glasses matched those of the corresponding Mg glasses, with the exception of glass Zn75. Here, no crystallisation exotherm was detected (Fig. 4f), and therefore no result for the processing window could be obtained. We have recently shown that replacing 2.5% of Ca with either Mg or Zn already resulted in a significantly widened processing window, while higher substitutions (up to 15%) did not cause any pronounced further improvement^27^. Composition Mg75 aside, the results presented here confirm this trend.

Heating microscopy gives insight into the shrinkage of powder compacts during heating, besides giving information about crystallisation and liquidus temperatures^37–39^. Bioglass 45S5 showed negligible shrinkage only (reduction in height about 3%). This is known to occur owing to crystallisation impeding viscous flow^8,9,38^. Interestingly, despite a significantly lower T_g_, sintering of Mg substituted glass Mg25 was not much more pronounced (height reduction 6% only). For further Mg for Ca substitution, sintering was improved much more, with 30 and 57% reduction in height for Mg50 and Mg75, respectively. In agreement with the trend observed for the processing window, complete substitution (Mg100) resulted in a lower reduction in sample height (17%) during thermal treatment, i.e. poorer sintering compared to the compositions containing calcium as well (Mg50 and Mg75). Sample height reduction for Zn glasses were relatively close to each other in comparison with the Mg series, with 16 (Zn25), 26 (Zn50), 20 (Zn75) and 27% (Zn100), respectively. Taken together, results show an improved high temperature processing of glass powder for both glass series, even for the fully substituted glasses, compared to 45S5.

Increasing substitution did not affect the beginning of sintering (T_si_), but heating microscopy curves showed a shift to lower liquidus temperatures with substitution. While 45S5 showed an onset of liquidus at about 1,110 °C, it decreased with increasing Mg substitution to 790 °C and with Zn substitution to 770 °C.

The starting temperature (T_xi_) of the crystallisation domain in heating microscopy did not show any significant effect with substitution, in agreement with relatively constant crystallisation onset temperature (T_c_) in DSC. By contrast, the end of the crystallisation domain (T_xf_) shifted to lower temperatures with increasing Mg or Zn substitution. As a result, the size of the crystallisation domain (Fig. 5b) of the glasses decreased almost linearly with increasing substitution, illustrating the increased resistance of the glasses against crystallisation. Comparing the two glass series, the crystallisation domains of the Zn glasses were smaller than those of the corresponding Mg glasses, with glass Zn100 revealing the smallest crystallisation domain. This suggests that Zn for Ca substitution affects the crystallisation behaviour of Bioglass 45S5 much more than Mg for Ca substitution. Interestingly, no such trend can be detected in the change of the processing window (Fig. 5a), where both series behaved in a very similar way. In general, the decreasing crystallisation tendency for both glass series can be attributed to various aspects. On the one hand, an increase in entropy owing to cation mixing promotes disorder in the glass structure and thus impedes crystallisation^40^. On the other hand, the substitution of Mg or Zn for Ca resulted in stronger chemical bonds owing to the higher field of those two cations. This also reduces the crystallisation tendency^40,41^.

**Figure 6:**
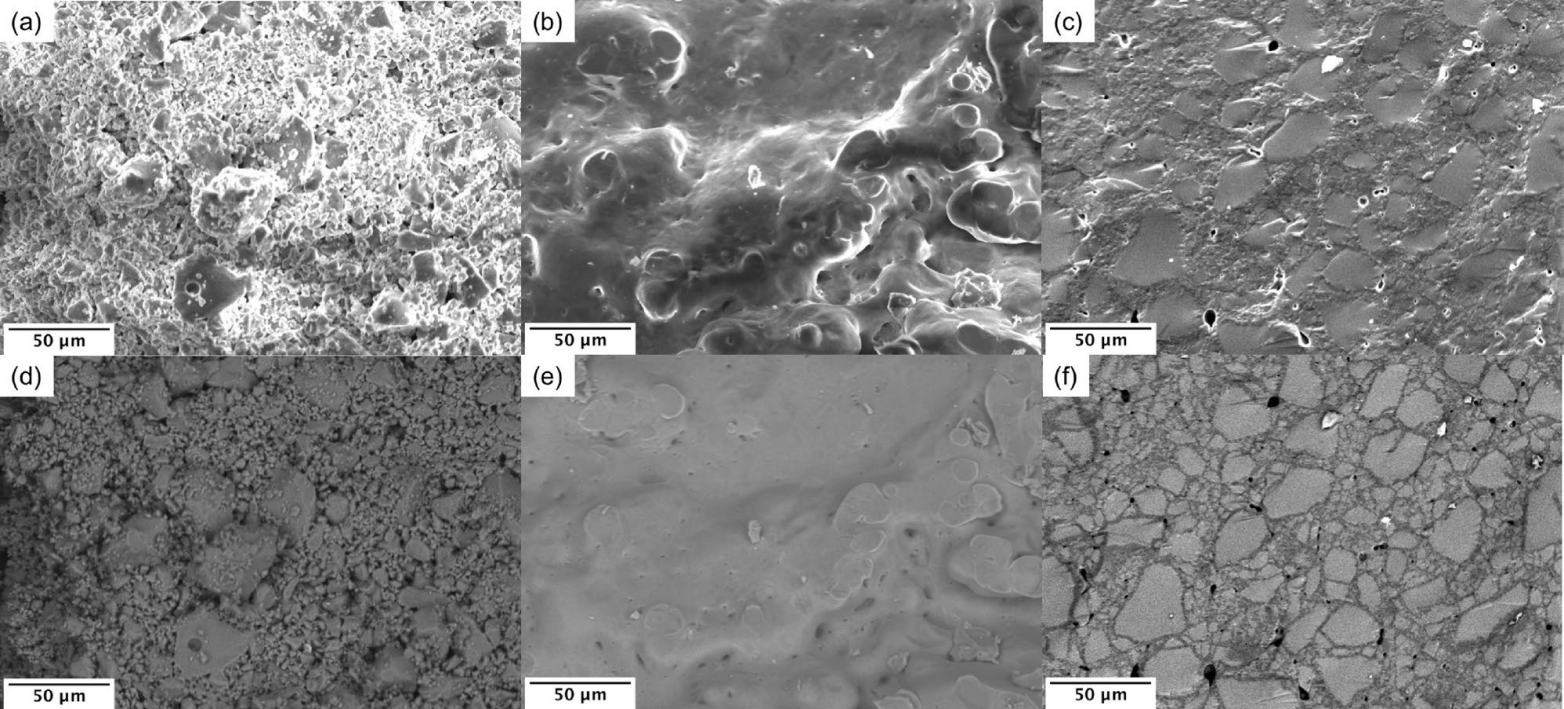
SEM images of freshly fractured sintered powder compacts of (**a**, **d**) 45S5, (**b**, **e**) Mg50 and (**c**, **f**) Zn50 with (**a**–**c**) images obtained in secondary electron and (**d**–**f**) corresponding backscattered electron mode images.

Results for particle size analysis for the glass powder used for the sintering and crystallisation studies are shown in Table 1. With the exception of glass Mg25, all glasses showed D50 values between 3.6 and 6.5 µm and D90 values below 25 µm. Results for Mg25 were slightly larger. SEM images of freshly fractured surfaces of sintered 45S5 powder compacts (Fig. 6a,d) show individual particles, which do not display any sintering neck formation between particles during heat treatment. This indicates that the 45S5 powder barely sintered together, in agreement with our previous results^8^. By contrast, Mg50 (Fig. 6b,e) has reached a high degree of sintering, in agreement with heating microscopy results: no individual particles can be identified in the SEM image and the surface looks homogeneous. Zn50 particles sintered together as well (Fig. 6c,f), but exhibited a different texture: many large (about 50 µm) angular particles as well as small particles (about 10 µm) are still present, possibly suggesting a bimodal particle size distribution. All particles are surrounded by a fine-grained matrix, that is probably crystalline as indicated by the darker colour in backscattered mode. Note the residual porosity of less than 1 vol% (black spots in SE and BSE mode).

**Table 1:** Glass particle distribution: cumulative volume percentage (D10, D50, D90, in µm) for glass powders.

Glass	D10	D50	D90
Mg100	1.4	6.5	19.1
Mg75	1.3	6.3	18.0
Mg50	1.1	5.0	16.7
Mg25	2.2	13.4	32.2
45S5	1.2	6.1	24.9
Zn25	1.2	6.4	21.8
Zn50	1.0	3.6	15.3
Zn75	1.1	5.1	15.3
Zn100	1.2	5.3	14.8

**Figure 7:**
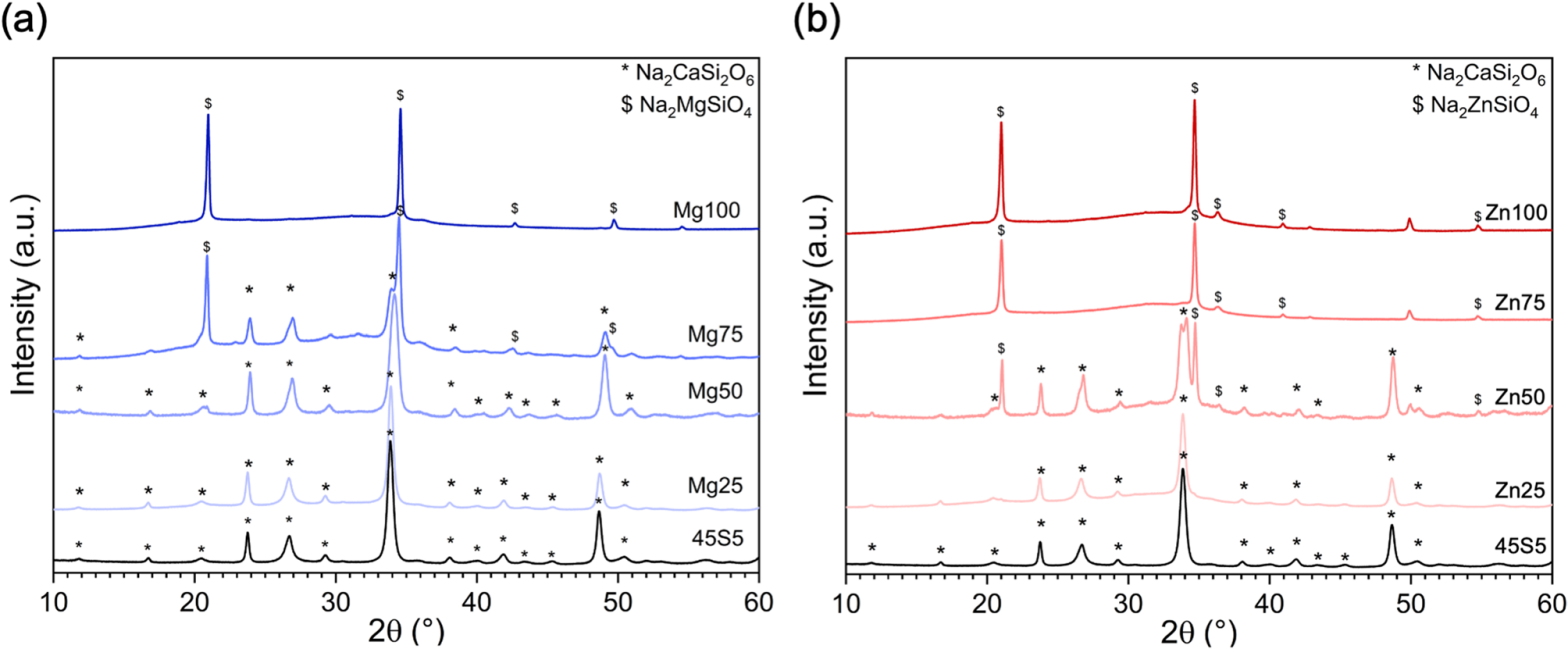
XRD patterns of sintered powder compacts of (**a**) Mg and (**b**) Zn substituted glasses.

XRD results confirmed crystallisation of all glasses during sintering (Fig. 7), including composition Mg50 which had sintered very well. This, again, indicates that crystallisation per se does not inhibit the dense sintering of bioactive glasses, as long as viscous flow is not inhibited^8^. Crystalline phases were silicate phases mostly. Bioglass 45S5 is known to show sodium calcium silicates as the main crystal phases during sintering at temperatures comparable to the ones employed here^38^. These sodium calcium silicate phases have previously been identified as either Na_2_CaSi_2_O_6_^36,42^ or Na_2_Ca_2_Si_3_O_9_^38,43,44^. Both show very similar diffraction patterns, are known to form solid-solutions within the series Na_6–2x_Ca_3+x_Si_6_O_18_ (0 ≤ x ≤ 1) and are similar to the mineral combeite^45,46^.

Interestingly, Mg or Zn for Ca substitution did not show a pronounced influence on the crystal phases formed even at up to 75% (Mg series, Fig. 7a) or 50% (Zn series, Fig. 7b) substitution. From compositions Mg75 and Zn50 a secondary phase appeared each, which was identified as a sodium magnesium silicate (cubic Na_2_MgSiO_4_; ICSD 00-019-1216, Inorganic Crystal Structure Database, FIZ Karlsruhe, Germany) and a sodium zinc silicate (e.g. Na_2_ZnSiO_4_; reference ICDS 00-037-0409 was for cubic Na_1.625_Zn_0.8125_Si_1.1875_O_4_), respectively. For the sodium zinc silicate, the reflex at about 21° 2θ is slightly shifted to lower 2θ values, which may be caused by a different Na/Zn ratio in the present crystal phase. For the sodium magnesium silicate, the lower intensity reflex at 24° 2θ is not present but may be hidden under the amorphous halo of the remaining glass phase. These two phases became the dominant crystal phases at higher substitution. For Mg100 an additional phase in minor concentration was detected, which could not be identified unambiguously. For Zn100 the additional minor phase could correspond to Zincite (cubic ZnO; ICSD 98-016-6360). These results suggest that phosphate remains in the glass upon crystallisation, which agrees with the low phosphate content.

We have recently shown that while crystallisation of sodium calcium silicate phases (e.g. Na_2_CaSi_2_O_6_ or Na_2_Ca_2_Si_3_O_9_)^45,46^ inhibits the dense sintering of Bioglass 45S5, it does not prevent it for glasses with lower T_g_ (e.g. through fluoride incorporation)^8^. This suggests that not only the type of crystal phase formed during sintering but also the relative positions of T_g_ and crystallisation temperatures to each other determine whether a glass sinters well or not. For Bioglass 45S5, viscous flow sintering is inhibited by an early onset of crystallisation^9,38^. The reduction in T_g_ for mixed Mg/Ca or Zn/Ca bioactive glass compositions, together with a lower overall tendency to crystallise is likely to be the reason for the improved sintering behaviour of the substituted glasses in the present study.

Taken together, these results suggest that using a combination of modifiers, particularly alkaline earths, like magnesium, or zinc, may be a promising approach for improving the sintering of Bioglass 45S5. By varying the actual amount of Mg or Zn in the glass, glasses with optimum therapeutic effects owing to the release of these ions during contact with physiological solutions may be obtained.

It is interesting to note that Mg and Zn for Ca substitution affect glass thermal properties in very similar ways. While this is easily explained by their similarity in ionic radius and field strength, previous results have shown that Mg and Zn affect glass degradation and ion release in aqueous media in very different ways^19,47^. While Mg for Ca substitution reduced ion release from the glass, Zn for Ca substitution nearly fully inhibited ion release at physiological pH^19^. Only at acidic pH, ion release profiles of the two series were more comparable. This was suggested to be caused by different structural roles of Mg and Zn in the glass, as discussed for glass density data above. However, further experiments, particularly structural analyses, are necessary to fully interpret the data.

## Methods

### Glass synthesis

Two glass series where calcium was replaced by increasing amounts of either magnesium or zinc (0–100%, Table 2) on a molar base were prepared by a melt-quench route as described previously^19^. Briefly, powders of SiO_2_, CaCO_3_, Na_2_CO_3_, NaPO_3_ and MgCO_3_ or ZnO were mixed and sintered together in a platinum crucible at 1,250 °C for 1 h and then melted for 1 h at 1,350 °C. Following the melting, the glasses were rapidly quenched into the water to avoid crystallization. The glasses were ground using a steel mortar and sieved using analytical sieves. Particle size distribution was determined by dynamic light scattering (DLS; Malvern Mastersizer 2000 equipped with a Hydro 2000S module). Glass cylinders for dilatometry analyses were prepared by re-melting the glass frit at 1,350 °C, pouring it into a brass mould, annealing in a preheated furnace (set to 30 K below glass transition temperature) and cooling to room temperature in the switched-off furnace overnight. Glass density was determined using helium pycnometry (AccuPyc 1330-1000, Micromeritics GmbH). Glass molar volume (V_m_) was calculated by dividing the molar weight of the glass by the experimental density, as described earlier^48^.

**Table 2:** Nominal glass compositions (mol%).

Glass	SiO_2_	P_2_O_5_	Na_2_O	CaO	ZnO	MgO
Mg100	46.1	2.6	24.4	–	–	26.9
Mg75	46.1	2.6	24.4	6.7	–	20.2
Mg50	46.1	2.6	24.4	13.45	–	13.45
Mg25	46.1	2.6	24.4	20.2	–	6.7
45S5	46.1	2.6	24.4	26.9	–	–
Zn25	46.1	2.6	24.4	20.2	6.7	–
Zn50	46.1	2.6	24.4	13.45	13.45	–
Zn75	46.1	2.6	24.4	6.7	20.2	–
Zn100	46.1	2.6	24.4	–	26.9	–

### Thermal characterisation

Glass transition (T_g_), defined as the inflection point of the transition temperature range, crystallisation onset (T_c_) and peak temperature (T_x_) were determined by differential scanning calorimetry (DSC; STA 449F1, Netzsch, 10 K min^−1^ up to 1,300 °C, particle size 125–250 µm). In addition, T_g_ and dilatometric softening point (T_d_) were measured by dilatometry (DIL 402 PC, Netzsch; 5 K min^−1^ up to 1,000 °C). Thermal expansion coefficients (TEC; 100–300 °C) were obtained from dilatometry curves. Theoretical values for TEC were calculated using the Appen model from the glass compositions. This model takes the individual TEC contribution of each glass component into account^34,49^. The glass processing window was calculated as the temperature range between T_g_ and the onset of crystallisation, as described earlier^22^.

Heating microscopy (HSM/ODHT, Misura Expert Systems; HSM, 5 K min^−1^) was used to measure changes in sample silhouette during heating. Characteristic temperatures such as beginning (i.e. onset) of sintering (T_so_) and end (i.e. offset) of sintering temperature (T_se_), start and the end temperature of crystallisation (T_xi,_ T_xf_) as well as liquidus temperature (T_f_) were recorded. Preparation of specimens for heating microscopy and subsequent analyses of curves were described previously^12^.

### Sintering and crystallization

For sintering and crystallisation experiments, powder compacts were prepared. 0.045 g of glass powder was filled into cylindrical moulds, two drops of ethanol were added and the powder was pressed by hand. Afterwards, another 0.045 g of powder was added and the procedure was repeated. After a waiting period of five minutes, the samples were removed from the mould. Powder compacts were heat treated in a furnace (HT 04/17, Nabertherm GmbH) to the offset of sintering temperature, corresponding to the following temperatures: 45S5 (606 °C), Mg25 (600 °C), Mg50 (602 °C), Mg75 (635 °C), Mg100 (603 °C), Zn25 (592 °C), Zn50 (595 °C), Zn75 (595 °C), Zn100 (615 °C), following the same heat treatment protocol as the one used in heating microscopy experiments (5 K min^−1^). After reaching the offset of sintering temperature, samples were removed from the furnace and air quenched. Uncoated, freshly fractured samples were visualised in a low-vacuum scanning electron microscope (SEM; JSM 6510LV, Jeol GmbH) using secondary electron or backscattered electron mode or ground and analysed using powder X-ray diffraction (XRD; Mini-flex 300, Rigaku Corporation, Tokio, Japan; Cu Kα, 30 kV, 20 mA, 10°–60° 2θ).
